# AFM-based Analysis of Wharton’s Jelly Mesenchymal Stem Cells

**DOI:** 10.3390/ijms20184351

**Published:** 2019-09-05

**Authors:** Renata Szydlak, Marcin Majka, Małgorzata Lekka, Marta Kot, Piotr Laidler

**Affiliations:** 1 Chair of Medical Biochemistry, Faculty of Medicine, Jagiellonian University Medical College, Kopernika 7, 31-034 Krakow, Poland; renata.szydlak@doctoral.uj.edu.pl; 2 Department of Transplantation, Institute of Pediatrics, Jagiellonian University Medical College, Wielicka 265, 30-663 Kraków, Poland; 3 Institute of Nuclear Physics, Polish Academy of Sciences, Radzikowskiego 152, 31-342 Kraków, Poland; 4 Department of Transplantation, Institute of Pediatrics, Jagiellonian University Medical College, Wielicka 265, 30-663 Kraków, Poland; 5 Chair of Medical Biochemistry, Faculty of Medicine, Jagiellonian University Medical College, Kopernika 7, 31-034 Krakow, Poland

**Keywords:** mesenchymal stem cells, WJ-MSCs, cell elasticity, actin cytoskeleton, atomic force microscopy, migration potential

## Abstract

Wharton’s jelly mesenchymal stem cells (WJ-MSCs) are multipotent stem cells that can be used in regenerative medicine. However, to reach the high therapeutic efficacy of WJ-MSCs, it is necessary to obtain a large amount of MSCs, which requires their extensive in vitro culturing. Numerous studies have shown that in vitro expansion of MSCs can lead to changes in cell behavior; cells lose their ability to proliferate, differentiate and migrate. One of the important measures of cells’ migration potential is their elasticity, determined by atomic force microscopy (AFM) and quantified by Young’s modulus. This work describes the elasticity of WJ-MSCs during in vitro cultivation. To identify the properties that enable transmigration, the deformability of WJ-MSCs that were able to migrate across the endothelial monolayer or Matrigel was analyzed by AFM. We showed that WJ-MSCs displayed differences in deformability during in vitro cultivation. This phenomenon seems to be strongly correlated with the organization of F-actin and reflects the changes characteristic for stem cell maturation. Furthermore, the results confirm the relationship between the deformability of WJ-MSCs and their migration potential and suggest the use of Young’s modulus as one of the measures of competency of MSCs with respect to their possible use in therapy.

## 1. Introduction

Mesenchymal stem cells (MSCs) represent a unique population of heterogeneous fibroblast-like cells. In culture conditions, MSCs adhere to the medium, have a high proliferative potential, and the ability to differentiate into different cell types, to regenerate and to repair tissues by secreting various growth factors and cytokines, and to cause an immunomodulatory effect on the immune system [1,2].

Those properties placed MSCs in the line of great interest amongst scientists and clinicians for a number of years. The possible use of MSCs as biological medicines may help to deliver new therapeutic alternatives in regenerative medicine and cell therapy.

Initially isolated, the most widely studied and used MSCs were mesenchymal stem cells collected from the bone marrow [3,4]. Later, these cells were isolated from a number of adult body tissues (e.g., adipose tissue, dental pulp, skeletal muscle, and others) [5]. Recently, the source of mesenchymal stem cells tested and used in various studies frequently originates from tissues related to pregnancy and child delivery (e.g., umbilical cord blood, umbilical cord, placenta). 

Wharton’s jelly (WJ) umbilical cord tissue was found to be a valuable source of mesenchymal stem cells. Human WJ-MSCs are multipotent, highly proliferating cells with a low immunogenic level making them a promising tool for tissue regeneration and repair [6]. The WJ-MSCs retrieval procedure is technically simpler than other sources and it is possible to isolate the rich population of MSCs efficiently, which does not raise any ethical controversy [7,8,9]. Therefore, these cells can also have a wide range of clinical applications in the treatment of many diseases, including cardiovascular disease, cancer, and nervous system disorders [10,11].

To obtain the high therapeutic efficacy of WJ-MSCs, it is necessary to dispose of large amounts of MSCs in vitro culture. Numerous studies have shown that in vitro expansion of MSCs can lead to changes in cellular behavior as cells lose their ability to proliferate, differentiate and migrate [12,13,14]. Methods of molecular biology, biochemistry, and genetics applied in previous studies have proven that prolonged in vitro cultivation increases the expression of cell cycle inhibitors such as APO, p21, p53, and p16INK4A [15,16,17]. Telomere shortening was also shown in each division, which is associated with the aging of the cell population [18]. Understanding the impact of the in vitro cultivation time on aging and progressive differentiation is a key factor in maintaining the therapeutic potential of MSCs. Therefore, it is necessary to look for new parameters describing changes of MSCs during cultivation, as well as optimization of isolation and cultivation methods that will enable MSCs to be obtained on an industrial scale in the future—in a repetitive, well-characterized manner, so that they always meet the required conditions and possess desirable feature (ability to adhere, migrate and release paracrine factors) without losing their original properties.

Recently, few studies have focused on the biomechanical properties of mesenchymal stem cells which regulate many processes such as proliferation, differentiation, and mobility necessary to achieve therapeutic efficacy [14,19,20]. The cytoskeleton, an internal scaffold of the cells and the main factor affecting mechanical features participates and regulates many processes, such as proliferation [21] differentiation [22], aging [23], adhesion [24], and migration [25], which are essential for the retention of cell function in vivo. The re-arrangement of actin microfilaments, among others, corresponds to the creation of essential processes necessary for movement as well as elasticity and change in the shape of cells. 

The biomechanical phenotype of the cell is related to its inherited properties like proliferation rate, internal molecular transport, signal transduction, adhesion, motility, and differentiation [21,22,24,25]. It is already well-known that mesenchymal stem cells, during the period of in vitro cultivation, lose their primary properties and cease to replicate and lose their differentiation capabilities. Consequently, studies on the correlation between biomechanical properties of mesenchymal stem cells and cellular functions could improve their characterization and lead to the identification of new potential biomarkers. 

The mechanical properties of cells can be quantified from measurements of cell deformability using, e.g., atomic force microscopy (AFM). This technique enables high-resolution measurements in a liquid environment, closer to the cells’ natural environment. The resulted measure of cell elasticity (Young’s modulus) reflects mainly the deformability of the cell cytoskeleton. Its value has been proven to be an excellent marker of oncogenic progression in various cancers [26], cardiovascular pathologies [27], cardiomyocyte aging [28] and skin aging [23] as well as stem cells differentiation [29]. For example, a study conducted by Maloney et al. showed that in the course of in vitro expansion of MSCs, the observed increase in the thickness of actin fibers accompanied the decrease in cell elasticity and simultaneously, enhanced their differentiation potential [19].

Regarding the possible use of MSCs for tissue regeneration, in addition to the need to obtain the appropriate number of therapeutic cells, one must carefully inspect their ability to migrate, which is critically essential concerning therapeutic goals. In practice, this means that when administered to the host organism, these cells would migrate specifically to sites of inflammation and damaged tissue typically associated with the outbreak of proinflammatory cytokines. Despite continued studies, the mechanism of migration of MSCs toward damages tissues has not been fully explained as yet and there is still a need to search for new solutions in studies on this field. It has been suggested that this process might be mediated by mechanisms similar to leukocyte migration, i.e., vascular endothelium rolling, endothelial adhesion, endothelial transmigration and tissue invasion [30,31]. It has also been shown that MSCs use a chemoattraction mechanism that drives them towards damaged tissues in response to inflammatory conditions [32,33,34,35]. Many studies have reported the functional expression of various chemokine receptors and adhesion molecules on human MSCs [31,36,37,38,39,40].

Mesenchymal cell motility is driven by polarized actin polymerization and depends on the organization of the F-actin cytoskeleton. The main factor regulating cell migration is the organization of the F-actin cytoskeleton, which also determines cellular deformability [41].

The studies carried out so far have proven that cellular deformability may be a marker of mobility and invasiveness in several types of cancer [42,43,44], but there is still lack of data focusing on the connection between MSCs elasticity and their migration potential. 

In our research, we focused on the nanomechanical characterization of WJ-MSCs during in vitro cultivation. The AFM measurements were carried out on mesenchymal stem cells routinely isolated from the Wharton’s jelly of 10 donors. Young’s modulus was determined for two indentation depths, i.e., for 300 nm and for 500 nm, using an approach described by Pogoda et al. [45]. The former depth describes quantitatively regions underlying the cell membrane that are rich in actin filaments. The latter concerns the mechanical response originating from deeply located internal organelles, including the cell nucleus. Our results show that mesenchymal stem cells changed their biomechanical properties during in vitro cultivation. To understand the meaning of such changes, we extended our research to determine if there was a correlation between the WJ-MSCs elasticity and their ability for transendothelial migration and invasion and in consequence their ability to home target tissues. Since pre-incubation with a pro-inflammatory cytokine has been shown to potentially increase the ability of MSCs to migrate, the magnitude of the transendothelial migration of WJ-MSCs pre-conditioned with pro-inflammatory cytokines was also measured. Migration tests were carried out in a modified Boyden chamber for two selected proinflammatory cytokines, i.e., IL-1β and IL-6.

The collected data suggests that Young’s modulus can be used to measure MSCs competency and is a biomarker for assessing their therapeutic potential. Our findings may contribute to the use of these cells within the clinical practice in the future.

## 2. Results

### 2.1. WJ-MSCs Manufacturing and Cell Characterization 

**Figure 1:**
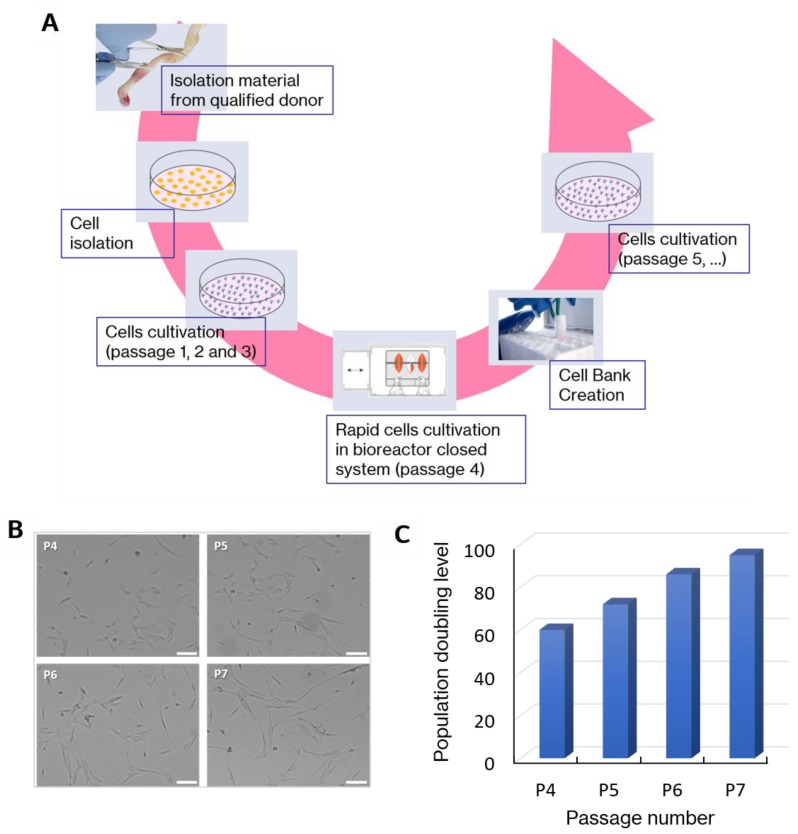
(**A**): The manufacturing process of mesenchymal stem cells—bases of the drug preparation. The steps of in vitro expansion of WJ-MSCs. (**B**): Representative optical microscopy images of WJ-MSCs at four different stage of in vitro cultivation (P4, P5, P6, P7). Some morphological changes were observed during long-term culture. Scalebar = 100 µm. (**C**): Population doubling level corresponding to passage number.

The steps of the in vitro expansion of WJ-MSCs are presented in Figure 1A. WJ-MSCs all had the morphology of adherent spindle-shaped cells resembling fibroblasts (Figure 1B). The population doubling level was been assigned to the corresponding passage number (Figure 1C).

**Figure 2:**
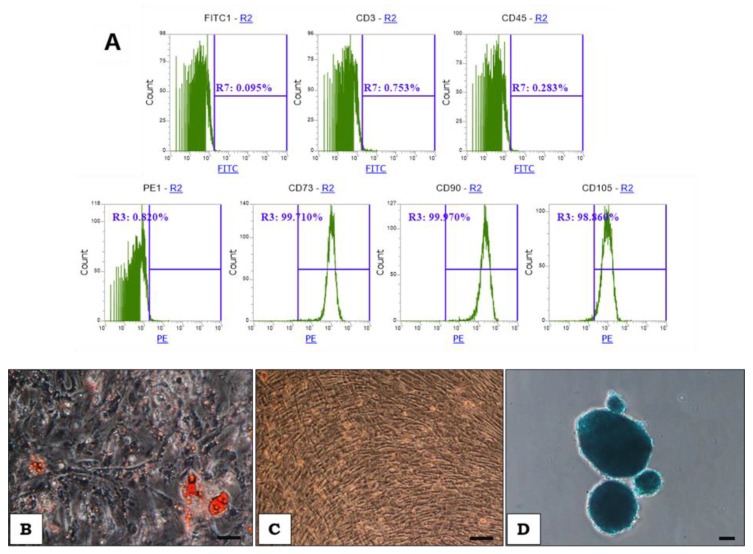
(**A**): The expression levels of surface markers on WJ-MSCs determined by flow cytometry. The WJ-MSCs are characterized by a high expression of CD73, CD90, CD105 (~90%) and a lack of expression of CD3 and CD45. (**B**, **C**, **D**): The trilineage potential of WJ-MSCs. WJ-MSCs were able to differentiate into adipocytes (B), osteoblasts (**C**) and chondrocyte (**D**). (**B**): intracellular lipid vacuoles visible in adipocytes after Oil Red staining; (**C**): osteoblasts (Alizarin Red staining); (**D**) chondrocytes (Alcian Blue staining); Light microscopy, magnification × 100, Scalebar = 100 µm.

Flow cytometry confirmed that the cells were positive for specific markers characteristic for MSCs, i.e., CD73, CD90, CD105 (~90%) and negative for CD3 and CD45 (Figure 2A). To verify the multipotent nature of isolated cells, differentiation assays were performed. The abilities of the cells to differentiate into adipocytes, osteoblasts, and chondrocytes were confirmed after about three weeks of differentiation (Figure 2B, C, D).

### 2.2. Deformability of WJ-MSCs Changes during in vitro Cultivation

**Figure 3:**
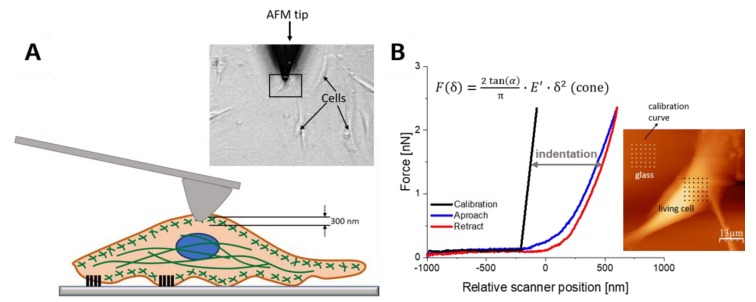
An idea of elasticity measurements using atomic force microscopy (AFM). (**A**): The illustration of the experimental method of AFM elasticity measurement on a living cell. (**B**): Force versus indentation curve was fitted with the Hertz—Sneddon mechanical model.

The deformability of mesenchymal stem cells was analyzed using AFM. Cells were cultured to 70% confluence. The elasticity of these cells was quantified at the indentation depth of 300 nm, corresponding to the mechanical response originating from actin-rich regions lying beneath the cell membrane and of 500 nm, including the response of various organelles, including cell nucleus (Figure 3). 

**Figure 4:**
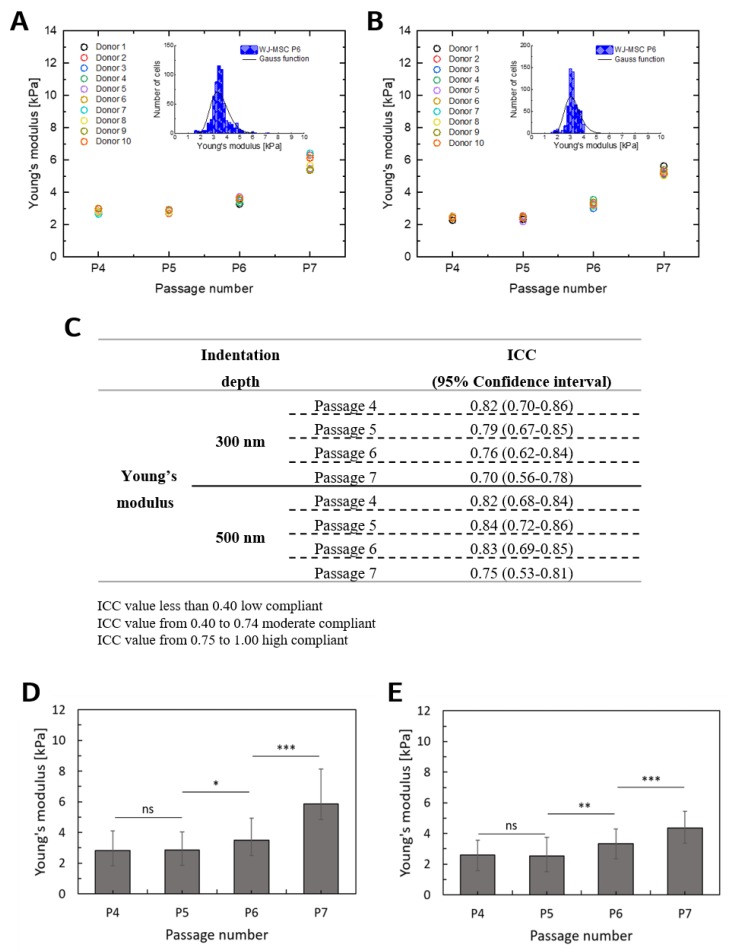
(**A**,**B**): The donor’s influence on the elasticity of WJ-MSCs from P4 to P7. Each donor is represented in the figures by a color. The results for each donor are presented as a mean determined for 60 cells. Young’s modulus calculated for indentation depths 300 nm (**A**) and 500 nm (**B**). (**C**): Intra-class correlations (ICC) obtained for all Young’s modulus determined for all participating donors. The values close to 0 indicate no compliance, while values close to 1 show perfect compatibility. (**D**, **E**): The elasticity of cells measured at various passage number (from P4 to P7), an average of 10 donors. The Young’s modulus is presented as a mean ± standard deviation determined for 600 cells from 21 600 sampling points. Young’s modulus calculated for indentation depths 300 nm (**D**) and 500 nm (**E**). An ANOVA test was applied to statistically verify the obtained results (ns: no significant difference).

The proliferation, differentiation and migration capacity of MSCs obtained from one donor might differ from the MSCs obtained from another donor [46], and a donor’s age is considered to affect the properties of MSCs [47]. MSCs origin may be crucial for potential use in cell-based therapies. Therefore, the present study aimed to characterize WJ-MSCs from different donors in order to investigate the elasticity and possible effects of donor variation on these cells (Figure 4A,B). To assess the repeatability and reproducibility of the elasticity measurements, the intra-class correlation coefficient was used, which takes values from 0 (not compliant) to 1 (perfect compatibility). No remarkable differences in Young’s modulus were noted for the WJ-MSCs taken from all participating donors. This indicates that the deformability of WJ-MSCs was not affected by donor variations (Figure 4C). Then, the average values of Young’s modulus of WJ-MSCs isolated from 10 donors were calculated (Figure 4D,E).

The elastic modulus of WJ-MSCs remained fairly constant for P4 and P5 for both indentation depths. The obtained values were approximately 2.8 kPa and 2.5 kPa for 300 nm and 500 nm, respectively. Significant increases (*p* < 0.05) were observed for P6 (3.50 ± 0.67 kPa) and P7 (5.84 ± 0.85 kPa) for an indentation depth of 300 nm and also for P6 (3.19 ± 0.77 kPa) and P7 (5.20 ± 0.60 kPa) for an indentation depth of 500 nm. These results are generally consistent with other studies [19,48,49,50], with differences attributed to a donor, AFM tip geometry, and location of indentation [51,52].

### 2.3. The F-actin Cytoskeleton is the Main Determinant of WJ-MSCs Deformability

**Figure 5:**
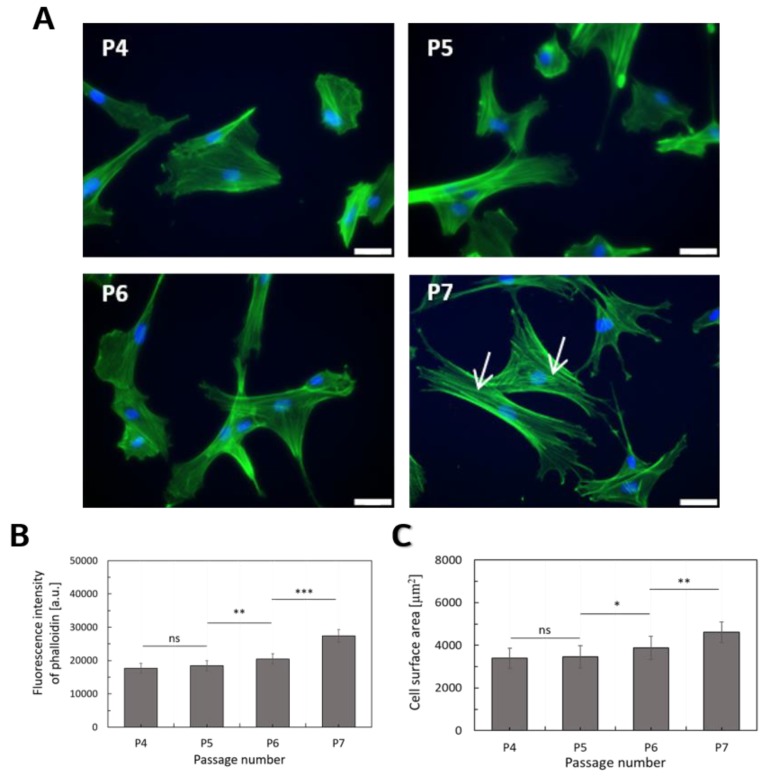
(**A**): Representative fluorescence microscopy images of WJ-MSCs at four different stage of in vitro cultivation (P4, P5, P6, P7). Some morphological changes were observed during long-term culture. Actin filament (green) distributions at four different stages of in vitro cultivation (P4, P5, P6, P7). The white arrows indicate thick, polymerized F-actin fibers. Phalloidin labeled with Alexa-Fluor 488 was used as a dye. Scalebar = 50 µm. (**B**): The change in phalloidin binding from P4 to P7 determined as a mean ± standard deviation and the average of 3 donors. Student’s t test was applied to statistically verify the obtained results (ns: no significant difference). (**C**): Cell surface area changes from P4 to P7 determined as a mean ± standard deviation for ~1000 cells, an average of 10 donors. The area occupied by single cells was determined by analyzing the cellular shape based on the fluorescence images of actin filaments. Student’s t-test was applied to statistically verify the obtained results (ns: no significant difference).

The morphology of WJ-MSCs was recorded at different stages of in vitro cultivation. The shape of single cells can be visualized by fluorescent staining of F-actin (Figure 5A). To follow the changes in the morphology of WJ-MSCs, fluorescence images were recorded for each passage after 4 days of growing. To quantify the morphological properties, the surface areas of the single cells were calculated from fluorescence images for cells taken at various stages of their cultivation (Figure 5C). To verify whether changes in the deformability of WJ-MSCs were accompanied by the different actin structures, fluorescence intensity measurements were employed. Actin filaments were stained with AlexaFluor488 conjugated with phalloidin. The analysis was performed for the same number of cells at real-time for each passage (Figure 5B).

During extended culture, cells showed signs of aging, including slowed proliferation, increased cell debris, and a change in appearance from spindle-shaped to a broad, flattened morphology (Figure 1B, Figure 5A), as previously described [13]. The surface area occupied by single cells was smaller in the case of WJ-MSCs at passages 4 and 5 (P4 and P5) as compared to WJ-MSCs at passage 7 (P7). It constituted 74% of the surface area calculated for cells at P7. For cells taken at P6, it was 83% of the surface area calculated for cells at P7. The lack of statistical significance verifying the difference between WJ-MSCs at P4 and P5 indicates the similar spreading capability of these cells. The obtained results show the highest fluorescence signal for WJ-MSCs taken at P7 and the lowest from the cells collected at P4 and P5. Analogously, as for spreading area, statistically significant differences in fluorescence intensity were observed for between cells at P5 and P6, as well as at P6 and P7. Based on these results, a distinct organization of F-actin in WJ-MSCs was postulated. A re-organization of actin filaments observed at different passages was also reflected in the alterations of the WJ-MSCs mechanical properties. Similar results were obtained by LeBlon et al., showing that changes in MSCs elasticity were related to the increases in actin stress fiber diameters [20].

**Figure 6:**
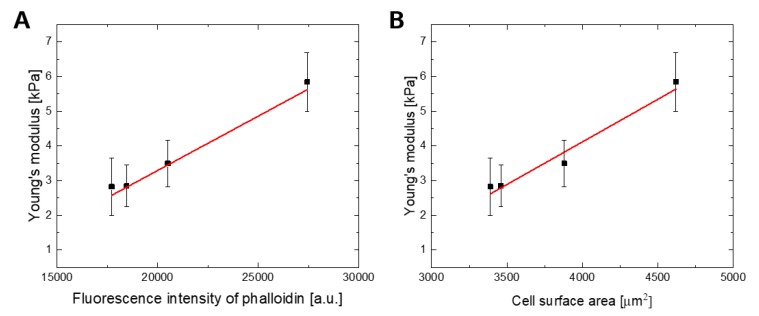
A linear relation on (**A**): Young’s modulus values of WJ-MSCs for an indentation depth of 300 nm and the fluorescence intensity of phalloidin (a goodness of the linear fit was quantified by the Pearson’s coefficient; Pearson’s r = 0.978), (**B**) Young’s modulus values of WJ-MSCs for an indentation depth of 300 nm and cell surface area (Pearson’s r = 0.981).

Finally, this study allowed the description of the relationship between observed changes in Young’s modulus and the amount of F-actin (Figure 6A) and also cell surface area (Figure 6B) using a linear function. 

### 2.4. Cellular Deformability is a Key Factor of WJ-MSCs Transmigration

**Figure 7:**
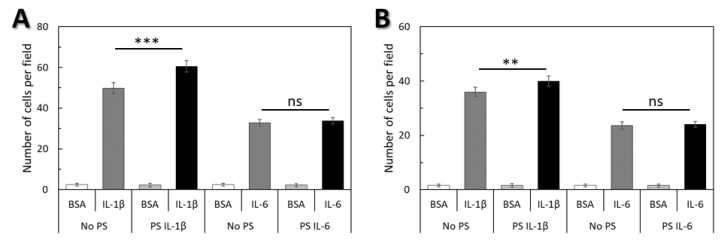
IL-1β and IL-6 induced WJ-MSCs transendothelial migration (**A**) and invasion (**B**). Pre-stimulation with IL-1β (PS IL 1β) increased transendothelial migration and invasion of WJ-MSCs toward IL-1β in vitro, but pre-stimulation with IL-6 (PS IL-6) did not affect the size of transendothelial migration and invasion toward IL-6. Data shown are the mean ± standard deviation of duplicate experiments, an average of 6 donors. An ANOVA with post-hoc Tukey test was applied to statistically verify the obtained results (ns: no significant difference).

To determine if there was a correlation between the deformability of WJ-MSCs and their ability for transendothelial migration (Figure 6A) and invasion (Figure 6B) migration tests were carried out in a modified Boyden chamber using pro-inflammatory cytokines IL-1β and IL-6. The quantitative migration assays of WJ-MSCs pre-conditioned with the same cytokines that were used as a chemoattractant were also performed (Figure 7).

Both IL-1β and IL-6 induced transendothelial migration and invasion of WJ-MSCs (Figure 6A,B). Migration toward IL-1β was significantly greater when compared with IL-6. When cells were pre-stimulated with IL-1β, migration toward IL-1β was significantly greater when compared with non-pre-stimulated migration toward IL-1β, but pre-stimulation with IL-6 had no effect on the migration of WJ-MSCs toward IL-6.

**Figure 8:**
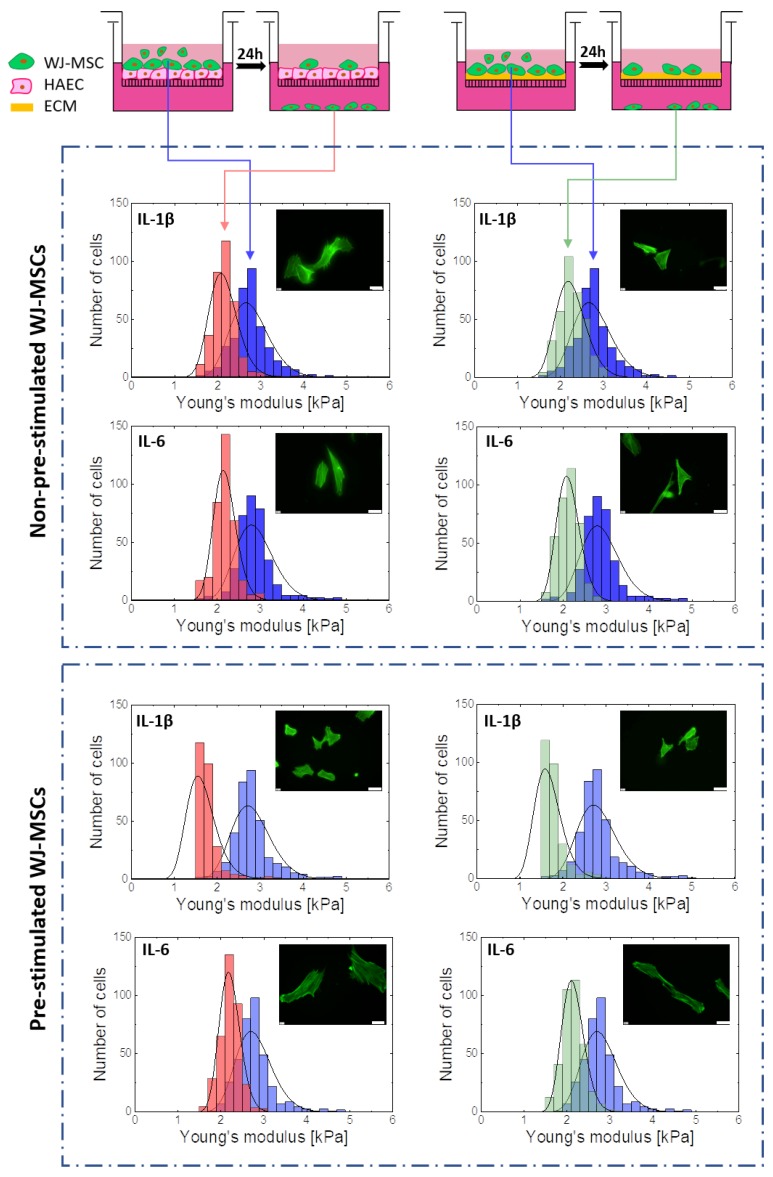
Comparison of the elasticity of WJ-MSCs before (blue) and after transendothelial migration (TEM) (red), and before (blue) and after the invasion (green) towards proinflammatory cytokines. The elasticity parameter distribution for 360 cells isolated from six donors. Young’s modulus calculated for indentation depths 300 nm. Gauss distribution fits (black solid curves). Insets—fluorescence images of subpopulation WJ-MSCs with migration potential (magnification 400 ×, Scalebar = 50 µm).

Cellular ability to deform is an important factor in cell migration to tissues. Therefore, an analysis of the elasticity of the subpopulation of the WJ-MSCs that were able to migrate across the endothelium and penetrate the basement membrane was performed. To prove the dominant role of cell deformability in WJ-MSCs transmigration, the elasticity of WJ-MSCs (P5) both before and after transendothelial migration and invasion assays was determined by AFM. Furthermore, a comparison of the elastic properties for both subsets using AFM was performed to evaluate the role of the susceptibility to mechanical distortions in the regulation of the WJ-MSCs migration and invasive potential (Figure 8).

The results show that the subpopulation of cells with migration potential was characterized by greater elasticity (Figure 8, red and green). In the case of WJ-MSCs showing abilities to migrate across the layer of endothelial cells and basement membrane, a significant narrowing of distributions and a shift of the center of gravity towards the lower values (Figure 8 red and green) were observed compared to the entire WJ-MSCs population. It is particularly worth noting that in the case of WJ-MSCs showing potential for transendothelial migration and invasion and preconditioned with IL-1β, an approximately 40% decrease of Young’s modulus was observed. Thus, the nanomechanical elasticity of the WJ-MSCs seems to be a primary determinant of their transmigration potential. 

## 3. Discussion

WJ-MSCs seems to be an excellent tool for future regenerative therapies of many diseases. There is a very low number of MSCs in the human body, usually no more than 0.01% of the total number of cells [53]. Due to their low number, the use of MSCs in regenerative medicine must base on their strong proliferative capacity in vitro, which allows us to obtain a large number of cells with comparable and adequate regenerative potential. The ability of MSCs to proliferate, differentiate and migrate, as well as to home target tissue to be treated, are key factors in cell therapy. All these processes are regulated with a significant contribution of the cytoskeleton. Differences in the cytoskeleton structure can be recently accurately quantified by measuring the mechanical properties of the cell.

AFM measurements of single-cell deformation is a unique approach that helps to understand the correlation between cell structure, mechanics and function [26]. Recent research increasingly suggests that biomechanical parameters may be just as important for characterizing cells as molecular markers [14]. These mechanical markers are mainly determined by the state of the cytoskeleton—a dynamic polymer network that regulates many key cell processes, including cell division, differentiation, mobility as well as intercellular interactions [21,22,24,25,54].

In our study, we used AFM to track changes in deformability at the level of the actin cytoskeleton and throughout the entire cell in the course of the multiplication of WJ-MSCs on an industrial scale, to the quantity that would allow them to be used in clinical practice. The MSCs were analyzed at various stages of in vitro cultures. The elasticities of MSCs were evaluated on the basis of the force-distance curve using AFM.

The measure of WJ-MSCs elasticity, Young’s modulus was found to be constant until P5 for both values of indentation depth. At higher passage numbers, we observed a statistically significant increase in the elasticity parameter (*p* < 0.05). These increases in cell elasticity were probably the result of the formation of stable actin filaments, correlated with the presence of stress fibers in cells (P7). Lebon et al. linked the increase in the value of the elasticity parameter to an increase in the diameter of the tensity fibers [20].

The dynamic structure of the cytoskeleton determines the morphology and mechanical properties of the cells. It is well-recognized that changes at the level of elasticity are associated with changes in cellular morphology. Microscopic images show that while most of the MSCs maintained their normal spindle shape in their early transit to P5, those in later stages showed less consistent cell morphologies and irregularly flattened geometry. In addition, the developed MSCs showed an increase in cell surface area, and hence, an increase in volume, resulting in a higher capacity value. It is believed that the increase in cell size is strongly associated with aging in vitro [55,56]. The lower values given for elasticity in P4 and P5 are probably related to the lack of actin stress fibers that appear in P6/P7. During long-term in vitro cultivation, MSCs lose the ability to divide and migrate, which may be due to the increase in MSCs surface area signaling the entry of cell into the maturation process. The life processes of smaller cells are more efficient because the substance exchange between the cytoplasm and the living environment is faster. The presented research shows that the increase of surface area was accompanied by an increase in stiffness. It seems that the observed increase of stiffness and surface area observed for the studied WJ-MSCs is a unique feature because so far, there are, in our knowledge no published results showing a correlation between cell size and stiffness. It is worth highlighting that the elasticity of cells, determined based on AFM measurements, strongly depends on the amount (density) and organization of actin filaments forming actin cytoskeleton inside the cells, while the cell spreading area is a result of adhesive interactions occurring within the contact area between cell and substrate surfaces. In the case of MSCs, previous researches demonstrated that the elasticity of cells was closely correlated with differentiation towards certain lines [20,29].

Our results also reveal that the elasticity of MSCs did not vary in reference to the donor. It is worth emphasizing that the MSCs population collected from each umbilical cord was homogeneous. A pure MSCs population is likely to have a better ability to proliferate, differentiate and secrete paracrine factors than a heterogeneous population consisting of MSCs and cells at different stages of differentiation. This shows that developed and introduced method of isolation and in vitro expansion of MSCs enables the achievement of a largely homogeneous population of cells, which is crucial for their therapeutic use. 

In most cases, MSCs are injected systemically. This demands their efficient migration and homing in a target tissue. The ability to direct migration MSCs towards a chemotactic stimulus and to actively penetrate the endothelium and extracellular matrix is crucial in the homing process to the place of expected destination. Furthermore, cellular deformability is an important factor in cell migration to tissues. The transendothelial migration and invasion potential of WJ-MSCs toward proinflammatory cytokines gradient showed a high level in response to IL-1β gradient. These findings are consistent with other reports [57,58]. We demonstrated that 30 min of exposure to IL-1β increased the migration of WJ-MSCs towards IL-1β in vitro. However, we did not observe this effect for cells treated with IL-6. It has previously been shown that IL-1β affects the properties of MSCs, and that several pathways are involved. IL-1β has been shown to induce WJ-MSCs migration via p38 MAPK signaling [58]. These findings indicate that an increase in the capacity of MSCs to home tissue can be achieved by modulating the mesenchymal stem cell response to various growth factors and cytokines.

Many studies have demonstrated that MSCs coordinate repair processes by several mechanisms, among which, the secretion of paracrine factors such as proinflammatory cytokines and growth factors appear to be key [59]. Moreover, MSCs secrete metalloproteinases that remodel the extracellular matrix in the scar tissue [60]. In the presented studies we assessed whether WJ-MSCs were able to migrate across the endothelium and basement membrane in response to inflammatory factors secreted by ischemic myocardium. In the next stage of research, it will be necessary to analyze the paracrine potential of homing WJ-MSCs.

In order to estimate the level of susceptibility to mechanical distortions in the regulation of transendothelial migration and invasion, the results obtained from transmigration assays and AFM measurements were carefully analyzed. The subpopulation of WJ-MSCs presenting migration potential are shown to demonstrate the increased deformability. Therefore, this suggests that there is a selective chemotactic migration of WJ-MSCs with higher deformability (lower Young’s modulus values). These results suggest that the elasticity of WJ-MSCs is related to the ability of these cells to move effectively in a selected environment. Thus, cellular deformability may be a useful biomarker for MSCs migration capacity and may help to create optimal strategies for MSCs proliferation in vitro. A similar correlation between cell elasticity and the migratory capability of cancerous cells was observed by Xu et al. [44] and Rudzka et al. [43]. Furthermore, a higher ability to deform may help cells to survive excessive mechanical loading that may occur in blood vessels or in damaged tissues.

In addition, the AFM measurements of cells with migration potential revealed an increase in the deformability of WJ-MSCs preconditioned with IL-1β, but the fluorescence images did not show changes in the F-actin cytoskeleton. Previous studies have shown that IL-1β may modulate cytoskeleton reorganization and regulate cell stiffness [61]. On the other hand, in studies conducted by Rudzka et al., it has been shown that migration through physical barriers, such as a porous filter, is possible due to increased MAPK signaling, which causes the re-organization of the actin cytoskeleton and increased susceptibility to tumor cell deformation [43]. Thus, it can be assumed that the mechanism of WJ-MSCs transmigration occurs in a similar manner and that the elasticity of WJ-MSCs is regulated by the MAPK pathway. Future research aimed to determine if p38 MAPK is also involved in cytoskeleton re-organization and deformability of WJ-MSCs is required. Considering the fact that for some cell types, cell stiffness is inversely correlated with migration rates [43,44], the microenvironment in which WJ-MSCs will be placed will increase their ability to deform and thus also increase their ability to penetrate deep into the tissues and consequently, the chance to home target tissue and regenerate its damaged parts.

Taken together, we demonstrated that WJ-MSCs at different stages of cultivation differed in their stiffness, size and actin organization. The F-actin cytoskeleton is the main determinant of WJ-MSCs elasticity. We assumed that stiffness is a suitable marker to trigger MSCs maturation and thus reduced functionality in clinical application, which might be useful to select or generate fully functional WJ-MSCs for cell-based therapies and tissue engineering applications. This study also shows that WJ-MSCs had a high invasive and transendothelial migration ability in response to inflammatory conditions (a gradient of proinflammatory cytokines that can be secreted by a damaged ischemic myocardium). In addition, the migration potential of WJ-MSCs was shown to be strongly correlated with cellular deformability of these cells, which was measured using an AFM. These results confirm the hypothesis that cellular deformability may be a useful biomarker for assessing the ability of WJ-MSCs to migrate towards damaged tissues in response to inflammatory agents. The current study provides valuable information for future research as well as clinical applications that could lead to the development of new MSCs-based therapeutic strategies to treat various diseases.

## 4. Materials and Methods 

### 4.1. Human WJ-MSCs Isolation and Cultivation

WJ-MSCs were isolated from human umbilical cords and cultured in compliance with the *Good Manufacturing Practice* (GMP) and all procedures were performed in line with *Good Laboratory Practice* (GLP). The umbilical cords were provided by The Polish Stem Cell Bank S.A. from 10 donors with Ethical Committee permissions required for the collection of human tissues as part of the project CIRCULATE (agreement no. Strategmed2/265761/10/NCBR/2015). Umbilical cords were collected into plastic tubes containing PBS buffer, supplemented with antibiotics and antimycotic, and then the tissue was mechanically cut into small fragments (25 mm^2^).

WJ-MSCs were isolated using an enzymatic digestion strategy. The isolated cells were cultured in Dulbecco’s modified Eagle’s medium (DMEM Low Glucose, Biowest, Riverside, MO, USA) supplemented with platelet lysate into tissue culture flasks at 37 °C, 5% CO_2,_ and 95% humidity. At confluence, the adherent cells were detached using Accutase cell detachment solution (BioLegend, San Diego, CA, USA) and re-seeded at tissue culture flasks and incubated again until confluence. After the third passage, WJ-MSCs were cultured in the Quantum Cell Expansion Bioreactor (Therumo BCT, Lakewood, CA, USA) and then the properties of WJ-MSCs at passage fourth were analyzed or cells were frozen in liquid nitrogen. For subsequent analysis, cells were thawed and grown in culture flasks at 37 °C, 5% CO_2_, and 95% humidity. 

### 4.2. Evaluation of Surface Markers Expression

The cells were harvested with Accutase (StemCells Inc., Newark, CA, USA), washed and incubated with fluorophore-labeled monoclonal antibodies (Becton Dickinson, Franklin Lakes, NJ, USA) for 30 min. at 4 °C in darkness. Next, the cells were washed with PBS to remove unbound antibodies and acquired using an Attune Nxt Flow Cytometer (Thermo Fisher Scientific, Waltham, MA, USA). A total of 10,000 viable cells (events) were analyzed per sample by use of Attune NxT Sofware v2.4.

### 4.3. Adipogenic, Osteogenic and Chondrogenic Differentiation 

The cells were seeded into 12-well plate at a density of 1.3 × 10^3^ cells/cm^2^ and cultured in the standard medium until the culture reached appropriate confluence. 

To evaluate the adipogenic potential, when the cells reached about 90% confluence, medium was replaced for complete MesenCultTM Adipogenic Differentiation Medium (StemCell Technologies, Vancouver, CA-BC, Canada). The medium was changed every 3 days. After 20 days of differentiation, the cells were fixed in 10% formalin for 20 min, washed in PBS and stained with Oil Red O (Sigma-Aldrich, St. Louis, MO, USA) according to standard procedure. 

The osteogenic differentiation was performed at 70%–80% confluence. The standard medium was replaced for complete MesenCult™ Osteogenic Differentiation (StemCell Technologies, Vancouver, CA-BC, Canada). The osteogenic medium was exchanged every 3–4 days for 18 days. When mineralization occurred, the cells were washed in PBS, fixed in 10% formalin for 20 min and stained with Alizarin Red (MERC, Darmstadt, Germany) to detect Ca^++^ deposits.

To assess the chondrogenic potential, the cells were cultured in standard conditions until they reached 90% confluence. Then, the standard medium was aspirated and replaced with complete MesenCult™-ACF Chondrogenic Differentiation Medium (StemCell Technologies, Vancouver, CA-BC, Canada). During 21 days of differentiation, the chondrogenic medium was changed every 3 days. The formation of cartilage proteoglycans was shown by Alcian Blue staining (Sigma-Aldrich, St. Louis, MO, USA).

### 4.4. Sample Preparation for AFM Measurements

WJ-MSCs from the fourth to the seventh passage were seeded on the plastic coverslips (Nunc Thermanox Coverslips, Thermo Fisher Scientific, Waltham, MA, USA) pre-coated with fibronectin and cultured until they reached 70% of confluency in a 5% CO_2_ atmosphere at 37 °C and 95% humidity. Prior to the AFM measurements, loosely bound cells were removed by rinsing the substrate with a fresh medium heated to 37 °C.

### 4.5. AFM Nanoindentation Measurements

The AFM used in our experiments was the XE-120 (from Park Systems, Suwon, South Korea) with a combined Olympus IX71 inverted optical microscope (Olympus, Tokyo, Japan) equipped with fluorescence. The microscope was used to move and align the probe above the cells. The measurements were accomplished using commercially available silicon nitride (Si_3_N_4_) cantilevers with the nominal spring constant of 0.02 N/m, open-angle of 36˚ and tip radius of 20 nm (OTR-4, Bruker, Camarillo, CA, USA). The measurements were conducted in cell culture media at room temperature.

Prior to the measurements, the cantilever was calibrated using the thermal fluctuations of the cantilevers according to Sader’s [62] with the resultant thermal spectrum fitted with Lorentzian function to determine the spring constant. The obtained spring value was 0.027 +/− 0.004 N/m. The cells were indented approximately over the nuclear region of individual cells. The force curves were recorded over a scan area of 25 μm^2^. A grid, 6 × 6 points, was set over the scanned region. On average, 36 force curves for every single cell were automatically recorded at the approach and retract velocities of 8 μm/s. The total number of measured cells for each donor was around 60 cells. The force curves were converted into a force versus indentation curves and further analyzed using Hertz contact mechanics with Sneddon’s approximations of the indenter geometry [63]. As in our case, the shape of the probing tip was a four-sided pyramid. For Young’s modulus determination, we assumed that the probe shape could be modeled as a cone. The average value of Young’s modulus was calculated by fitting the Gaussian function to the histogram of the values obtained for each curve.

### 4.6. Fluorescence Staining

In order to visualize F-actin in cell cytoskeleton, WJ-MSCs were cultured on the fibronectin-coated plastic coverslips were fixed in 3.7% paraformaldehyde for 20 min at room temperature. Cold 0.2% Triton X-100 was used for cells permeabilization (5 min), followed by rinsing them with PBS buffer (3 ×  2 min). The cells were incubated for 45 min at room temperature with Alexa Fluor 488 (absorption maximum 488 nm—blue light, and emission 518 nm—green) conjugated with phalloidin (0.033 μM in PBS, Molecular Probes, Eugene, OR, USA) to enable the visualization of F-actin and washed again with PBS (3  × 2 min). Nuclear DNA was counterstained using the Hoechst solution (the absorption at 355 nm—ultraviolet, and the emission at 465 nm—blue) (1 mg/mL in PBS, Sigma-Aldrich, St. Louis, MO, USA) for 15 min, followed by washing in PBS (3  ×  2 min).

### 4.7. Image Recording

All the fluorescence images were taken using an optical Olympus IX51 microscope equipped with a 100-W Mercury light source (U-LH100HG, Olympus, Tokyo, Japan). For actin filaments, visualization U-MWIG2 filter (*λexit* = 530−550 nm, *λemit* = 590 nm) was used, and U-MNB2 one (*λexit* = 470−490 nm, *λemit* = 520 nm) was used to detect fluorescently labeled cell nuclei. Fluorescence images were recorded using the XC10 digital camera (Olympus). The maximum resolution of the captured image by this camera was 2080 ×  1544 px. All the images were recorded using CellSense Dimensions (Olympus) software with the objective 20 × (Universal Plan Fluorite, the magnification of 200).

### 4.8. Cell Spreading Area Determination

To quantify the change of WJ-MSCs morphology of single cells, fluorescence images of actin cytoskeleton were used to calculate an effective surface area of a single cell, according to the procedure described previously by Prauzner-Bechcicki et al. [64]. These calculations were processed using an open-source code “ImageJ” (http://rsbweb.nih.gov/ij/) (access on 16.12.2018). 

### 4.9. F-actin Content Determination

To determine the content of F-actin, cells taken at the passage from 4 to 7 were seeded in black-walled 24-well plates with a density of 5 000 cells/well. After 72 h cells were fluorescence stained with Alexa Fluor 488 conjugated with phalloidin (0.033 μM in PBS, Molecular Probes, Eugene, OR, USA). Phalloidin binding was quantified with a SpectraMax Gemini microplate reader (Molecular Devices, Sunnyvale, CA, USA) using the appropriate excitation and emission filters for the Alexa Fluor 488 fluorophores: Ex488/Em518.

### 4.10. Transmigration Assays

Cell migration across the endothelium was analyzed by a modified Boyden‘s chamber with 8-μm pore polycarbonate membrane inserts (Costar^®^ Transwell^®^, Corning, Kennebunk, ME, USA). HAEC (seeding density 5 × 10^4^ cells/insert) were first cultured for 48 h on the upper surface of the filter and grown in EGM-2 BulletKit medium (Lonza Inc., Walkersville, MD, USA) until confluence in a humidified atmosphere (37 °C, 5% CO_2_). 

Cell invasion was analyzed using the Corning BioCoat Matrigel Invasion Chambers with 8-μm pore polycarbonate membrane inserts and a thin layer of Matrigel basement membrane matrix (Cat.# 354480, Corning, Bedford, MA, USA), according to the manufacturer’s protocol.

Prior to use in transmigration assay, WJ-MSCs were labeled with a fluorescent dye (2.5 μM, calcein AM, BD Biosciences) at 37 °C for 30 min. Cells were washed twice with PBS and resuspended in the growth medium. In the selected experiments, WJ-MSCs were co-incubated with IL-1β (50 ng/mL, PeproTech EC Ltd., London, UK) or IL-6 (50 ng/mL, PeproTech EC Ltd., London, UK) for 30 min.

Then, WJ-MSCs (each 5 × 10^4^ per well) were placed into upper chambers. Lower chambers were filled with medium contained IL-1β (50 ng/mL) or IL-6 (50 ng/mL) was placed. Medium containing 0.5% BSA or 10% FBS was the negative and positive control, respectively.

After 24 h of co-culture, HAEC monolayers and nonmigrating WJ-MSCs on the upper surface of the membrane were removed using a cotton swab. The number of cells on the lower part of the filter was counted in five randomly selected visual fields using fluorescence microscopy (Olympus Corporation, Tokyo, Japan). The experiments were repeated twice.

### 4.11. Statistical Significance

A statistical analysis was performed using STATISTICA 12 software (StatSoft Inc., Tulsa, OK, USA). All values are expressed as a mean ± SEM. Statistical significance was identified by ANOVA or t-tests to compare means. Additionally, intra-class correlations (ICC), based on the mixed models, were calculated to estimate the effect of the donor on cells elastic properties. 

## 5. Conclusions

Human MSCs subjected to intense development in vitro may be subject to morphological and biomechanical changes. These properties are also regulated by changes in the actin cytoskeleton architecture. AFM measurements can be used to complement standard procedures for assessing the therapeutic capacity of MSCs, offering a goal, an innovative and quantitative diagnostic approach allowing an unbiased evaluation of the experiments.

## Abbreviations

AFM	Atomic Force Microscopy
GMP	Good Manufacturing Practice
GLP	Good Laboratory Practice
MSCs	Mesenchymal Stem Cells
WJ-MSCs	Wharton’s Jelly Mesenchymal Stem Cells

## References

[1] Da Silva Meirelles L. (2006). Mesenchymal stem cells reside in virtually all post-natal organs and tissues. J. Cell Sci..

[2] da Silva Meirelles L., Fontes A.M., Covas D.T., Caplan A.I. (2009). Mechanisms involved in the therapeutic properties of mesenchymal stem cells. Cytokine Growth Factor Rev..

[3] Friedenstein A.J., Petrakova K.V., Kurolesova A.I., Frolova G.P. (1968). Heterotopic of Bone, Marrow. Analysis of precursor cells for osteogenic and hematopoietic tissues. Transplantation.

[4] Pittenger M.F., Mackay A.M., Beck S.C., Jaiswal R.K., Douglas R., Mosca J.D., Moorman M.A., Simonetti D.W., Craig S., Marshak D.R. (1999). Multilineage potential of adult human mesenchymal stem cells. Science.

[5] Berebichez-Fridman R., Montero-Olvera P.R. (2018). Sources and clinical applications of mesenchymal stem cells state-of-the-art review. Sultan Qaboos Univ. Med. J..

[6] Jin H.J., Bae Y.K., Kim M., Kwon S.J., Jeon H.B., Choi S.J., Kim S.W., Yang Y.S., Oh W., Chang J.W. (2013). Comparative analysis of human mesenchymal stem cells from bone marrow, adipose tissue, and umbilical cord blood as sources of cell therapy. Int. J. Mol. Sci..

[7] Nagamura-Inoue T. (2014). Umbilical cord-derived mesenchymal stem cells: Their advantages and potential clinical utility. World J. Stem Cells.

[8] Batsali A.K., Batsali A., Kastrinaki M.-C.A., Papadaki H., Pontikoglou C. (2013). Mesenchymal Stem Cells Derived from Wharton's Jelly of the Umbilical Cord: Biological Properties and Emerging Clinical Applications. Curr. Stem Cell Res. Ther..

[9] Moretti P., Hatlapatka T., Marten D., Lavrentieva A., Majore I., Hass R., Kasper C. (2010). Mesenchymal Stromal Cells Derived from Human Umbilical Cord Tissues: Primitive Cells with Potential for Clinical and Tissue Engineering Applications. Adv. Biochem. Eng. Biotechnol..

[10] Majka M., Sułkowski M., Badyra B., Musiałek P. (2017). Concise review: Mesenchymal stem cells in cardiovascular regeneration: Emerging research directions and clinical applications. Stem Cells Translational Med..

[11] Dmitrieva R.I., Minullina R., Bilibina A.A., Tarasova O.V., Anisimov S.V., Zaritskey A.Y. (2012). Bone marrow- and subcutaneous adipose tissue-derived mesenchymal stem cells: Differences and similarities. Cell Cycle.

[12] Trounson A., McDonald C. (2015). Stem Cell Therapies in Clinical Trials: Progress and Challenges. Cell Stem Cell..

[13] Yang Y.H.K., Ogando C.R., Wang S.C., Chang T.Y., Barabino G.A. (2018). Changes in phenotype and differentiation potential of human mesenchymal stem cells aging in vitro. Stem Cell Res. Ther..

[14] McGrail D.J., McAndrews K.M., Dawson M.R. (2013). Biomechanical analysis predicts decreased human mesenchymal stem cell function before molecular differences. Exp. Cell Res..

[15] Tsai C.C., Chen Y.J., Yew T.L., Chen L.L., Wang J.Y., Chiu C.H., Hung S.C. (2011). Hypoxia inhibits senescence and maintains mesenchymal stem cell properties through down-regulation of E2A-P21 by HIF-TWIST. Blood.

[16] Boregowda S.V., Krishnappa V., Chambers J.W., Lograsso P.V., Lai W.T., Ortiz L.A., Phinney D.G. (2012). Atmospheric oxygen inhibits growth and differentiation of marrow-derived mouse mesenchymal stem cells via a P53-dependent mechanism: Implications for long-term culture expansion. Stem Cells.

[17] Shibata K.R., Aoyama T., Shima Y., Fukiage K., Otsuka S., Furu M., Kohno Y., Ito K., Fujibayashi S., Neo M. (2007). Expression of the P16INK4A gene is associated closely with senescence of human mesenchymal stem cells and is potentially silenced by DNA methylation during in vitro expansion. Stem Cells.

[18] Baxter M.A. (2004). Study of telomere length reveals rapid aging of human marrow stromal cells following in vitro expansion. Stem Cells.

[19] Maloney J.M., Nikova D., Lautenschläger F., Clarke E., Langer R., Guck J., Van Vliet K.J. (2010). Mesenchymal Stem cell mechanics from the attached to the suspended state. Biophys. J..

[20] LeBlon C.E., Casey M.E., Fodor C.R., Zhang T., Zhang X., Jedlicka S.S. (2015). Correlation between in vitro expansion-related cell stiffening and differentiation potential of human mesenchymal stem cells. Differentiation.

[21] Fletcher D.A., Mullins R.D. (2010). Cell mechanics and the cytoskeleton. Nature..

[22] Ambriz X., de Lanerolle P., Ambrosio J.R. (2018). The mechanobiology of the actin cytoskeleton in stem cells during differentiation and interaction with biomaterials. Stem Cells Int..

[23] Dulińska-Molak I., Pasikowska M., Pogoda K., Lewandowska M., Eris I., Lekka M. (2014). Age-related changes in the mechanical properties of human fibroblasts and its prospective reversal after anti-wrinkle tripeptide treatment. Int. J. Pept. Res. Ther..

[24] Parsons J.T., Horwitz A.R., Schwartz M.A. (2010). Cell adhesion: Integrating cytoskeletal dynamics and cellular tension. Nature Rev. Mol. Cell Biol..

[25] Tang D.D., Gerlach B.D. (2017). The roles and regulation of the actin cytoskeleton, intermediate filaments and microtubules in smooth muscle cell migration. Respir. Res..

[26] Zemła J., Danilkiewicz J., Orzechowska B., Pabijan J., Seweryn S., Lekka M. (2018). Atomic force microscopy as a tool for assessing the cellular elasticity and adhesiveness to identify cancer cells and tissues. Semin. Cell Dev. Biol..

[27] Rianna C., Radmacher M. Cell mechanics as a marker for diseases: Biomedical applications of AFM. AIP Conference Proceedings, Penang, Malaysia, 26 September 2016.

[28] Lieber S.C., Vatner S.F., Aubry N., Diaz G., Pain J., Kim S.-J. (2004). Aging increases stiffness of cardiac myocytes measured by atomic force microscopy nanoindentation. Am. J. Physiol. Circ. Physiol..

[29] Han I., Kwon B.S., Park H.K., Kim K.S. (2017). Differentiation potential of mesenchymal stem cells is related to their intrinsic mechanical properties. Int. Neurourol. J..

[30] Nitzsche F., Müller C., Lukomska B., Jolkkonen J., Deten A., Boltze J. (2017). Concise review: MSC adhesion cascade—insights into homing and transendothelial migration. Stem Cells.

[31] Rüster B., Göttig S., Ludwig R.J., Bistrian R., Müller S., Seifried E., Gille J., Henschler R. (2006). Mesenchymal stem cells display coordinated rolling and adhesion behavior on endothelial cells. Blood.

[32] Berry M.F., Engler A.J., Joseph W.Y., Pirolli T.J., Bish L.T., Jayasankar V., Morine K.J., Gardner T.J., Discher D.E., Lee S.H. (2006). Regulation and function of stem cells in the cardiovascular system mesenchymal stem cell injection after myocardial infarction improves myocardial compliance. Am. J. Physiol. Hear. Circ. Physiol..

[33] Fox J.M., Chamberlain G., Ashton B.A., Middleton J. (2007). Recent advances into the understanding of mesenchymal stem cell trafficking. Brit. J. Haematol..

[34] Sohni A., Verfaillie C.M. (2013). Mesenchymal stem cells migration homing and tracking. Stem Cells Int..

[35] Wang S., Wu Y., Atkinson K. (2016). The role of chemokines in mesenchymal stromal cell homing to sites of inflammation, including infarcted myocardium. The Biology and Therapeutic Application of Mesenchymal Cells.

[36] Ip J.E., Wu Y., Huang J., Zhang L., Pratt R.E., Dzau V.J. (2007). Mesenchymal stem cells use integrin 1 NOT CXC chemokine receptor 4 for myocardial migration and engraftment. Mol. Biol. Cell.

[37] Steingen C., Brenig F., Baumgartner L., Schmidt J., Schmidt A., Bloch W. (2008). Characterization of key mechanisms in transmigration and invasion of mesenchymal stem cells. J. Mol. Cell. Cardiol..

[38] Kia N.A., Bahrami A.R., Ebrahimi M., Matin M.M., Neshati Z., Almohaddesin M.R., Aghdami N., Bidkhori H.R. (2011). comparative analysis of chemokine receptor’s expression in mesenchymal stem cells derived from human bone marrow and adipose tissue. J. Mol. Neurosci..

[39] Honczarenko M., Le Y., Swierkowski M., Ghiran I., Glodek A.M., Silberstein L.E. (2005). Human bone marrow stromal cells express a distinct set of biologically functional chemokine receptors. Stem Cells.

[40] Ringe J., Strassburg S., Neumann K., Endres M., Notter M., Burmester G.R., Kaps C., Sittinger M. (2007). Towards in situ tissue repair: Human mesenchymal stem cells express chemokine receptors CXCR1, CXCR2 and CCR2, and migrate upon stimulation with CXCL8 but Not CCL2. J. Cell Biochem..

[41] Olson M.F., Sahai E. (2009). The actin cytoskeleton in cancer cell motility. Clin. Exp. Metastasis.

[42] Iwanicki M.P., Davidowitz R.A., Ng M.R., Besser A., Muranen T., Merritt M., Danuser G., Ince T., Brugge J.S. (2011). Ovarian cancer spheroids use myosin-generated force to clear the mesothelium. Cancer Discov..

[43] Rudzka D.A., Spennati G., McGarry D.J., Chim Y.-H., Neilson M., Ptak A., Munro J., Kalna G., Hedley A., Moralli D. (2019). Migration through physical constraints is enabled by MAPK-induced cell softening via actin cytoskeleton re-organization. J. Cell Sci..

[44] Xu W., Mezencev R., Kim B., Wang L., McDonald J., Sulchek T. (2012). Cell stiffness is a biomarker of the metastatic potential of ovarian cancer cells. PLoS ONE.

[45] Pogoda K., Jaczewska J., Wiltowska-Zuber J., Klymenko O., Zuber K., Fornal M., Lekka M. (2012). Depth-sensing analysis of cytoskeleton organization based on AFM data. Eur. Biophys. J..

[46] Phinney D.G., Kopen G., Righter W., Webster S., Tremain N., Prockop D.J. (1999). Donor variation in the growth properties and osteogenic potential of human marrow stromal cells. J. Cell. Biochem..

[47] Beane O.S., Fonseca V.C., Cooper L.L., Koren G., Darling E.M. (2014). Impact of aging on the regenerative properties of bone marrow-, muscle-, and adipose-derived mesenchymal stem/stromal cells. PLoS ONE.

[48] Titushkin I., Cho M. (2007). Modulation of cellular mechanics during osteogenic differentiation of human mesenchymal stem cells. Biophys. J..

[49] Yourek G., Hussain M.A., Mao J.J. (2007). Cytoskeletal changes of mesenchymal stem cells during differentiation. ASAIO J..

[50] Docheva D., Padula D., Popov C., Mutschler W., Clausen-Schaumann H., Schieker M. (2008). Researching into the cellular shape, volume and elasticity of mesenchymal stem cells, osteoblasts and osteosarcoma cells by atomic force microscopy: stem cells. J. Cell. Mol. Med..

[51] Schillers H., Rianna C., Schäpe J., Luque T., Doschke H., Wälte M., Uriarte J.J., Campillo N., Michanetzis G.P.A., Bobrowska J. (2017). Standardized Nanomechanical atomic force microscopy procedure (SNAP) for measuring soft and biological samples. Sci. Rep..

[52] Rico F., Roca-Cusachs P., Gavara N., Farré R., Rotger M., Navajas D. (2005). Probing mechanical properties of living cells by atomic force microscopy with blunted pyramidal cantilever tips. Phys. Rev. E.

[53] Banfi A., Muraglia A., Dozin B., Mastrogiacomo M., Cancedda R., Quarto R. (2000). Proliferation kinetics and differentiation potential of ex vivo expanded human bone marrow stromal cells: Implications for their use in cell therapy. Exp. Hematol..

[54] Bershadsky A.D., Balaban N.Q., Geiger B. (2003). Adhesion-dependent cell mechanosensitivity. Annu. Rev. Cell Dev. Biol..

[55] Wexler S.A., Donaldson C., Denning-Kendall P., Rice C., Bradley B., Hows J.M. (2003). adult bone marrow is a rich source of human mesenchymal “stem” cells but umbilical cord and mobilized adult blood are not. Br. J. Haematol..

[56] Hayflick L. (1989). Antecedents of cell aging research. Exp. Gerontol..

[57] Chen M.S., Lin C.Y., Chiu Y.H., Chen C.P., Tsai P.J., Wang H.S. (2018). IL-1β-Induced matrix metalloprotease-1 promotes mesenchymal stem cell migration via PAR1 and G-protein-coupled signaling pathway. Stem Cells Int..

[58] Guo Y.C., Chiu Y.H., Chen C.P., Wang H.S. (2018). Interleukin-1β induces CXCR3-mediated chemotaxis to promote umbilical cord mesenchymal stem cell transendothelial migration. Stem Cell Res. Ther..

[59] Pankajakshan D., Agrawal D.K. (2014). Mesenchymal stem cell paracrine factors in vascular repair and regeneration. J. Biomed. Technol. Res..

[60] Molina E.J., Palma J., Gupta D., Torres D., Gaughan J.P., Houser S., Macha M. (2009). Reverse remodeling is associated with changes in extracellular matrix proteases and tissue inhibitors after mesenchymal stem cell (MSC) treatment of pressure overload hypertrophy. J. Tissue Eng. Regen. Med..

[61] Qi J., Fox A.M., Alexopoulos L.G., Chi L., Bynum D., Guilak F., Banes A.J. (2006). IL-1β decreases the elastic modulus of human tenocytes. J. Appl. Physiol..

[62] Sader J.E., Larson I., Mulvaney P., White L.R. (1995). Method for the calibration of atomic force microscope cantilevers. Rev. Sci. Instrum..

[63] Lekka M., Laidler P., Ignacak J.J., Labd M., Lekki J., Struszczyk H., Stachura Z., Hrynkiewicz A.Z. (2001). The effect of chitosan on stiffness and glycolytic activity of human bladder cells. Biochim. Biophys. Acta Mol. Cell Res..

[64] Prauzner-Bechcicki S., Raczkowska J., Madej E., Pabijan J., Lukes J., Sepitka J., Rysz J., Awsiuk K., Bernasik A., Budkowski A. (2015). PDMS Substrate stiffness affects the morphology and growth profiles of cancerous prostate and melanoma cells. J. Mech. Behav. Biomed. Mater..

